# Improved methods for haemozoin quantification in tissues yield organ-and parasite-specific information in malaria-infected mice

**DOI:** 10.1186/1475-2875-11-166

**Published:** 2012-05-14

**Authors:** Katrien Deroost, Natacha Lays, Sam Noppen, Erik Martens, Ghislain Opdenakker, Philippe E Van den Steen

**Affiliations:** 1 Laboratory of Immunobiology, Rega Institute, University of Leuven, Leuven, Belgium; 2 Laboratory of Molecular Immunology, Rega Institute, University of Leuven, Leuven, Belgium; 3 Currently at the laboratory of Virology and Chemotherapy, Rega Institute, University of Leuven, Leuven, Belgium

**Keywords:** Chemo-luminescence, Densitometry, Haemozoin quantification, Malaria pigment, *Plasmodium*

## Abstract

**Background:**

Despite intensive research, malaria remains a major health concern for non-immune residents and travelers in malaria-endemic regions. Efficient adjunctive therapies against life-threatening complications such as severe malarial anaemia, encephalopathy, placental malaria or respiratory problems are still lacking. Therefore, new insights into the pathogenesis of severe malaria are imperative. Haemozoin (Hz) or malaria pigment is produced during intra-erythrocytic parasite replication, released in the circulation after schizont rupture and accumulates inside multiple organs. Many *in vitro* and *ex vivo* immunomodulating effects are described for Hz but *in vivo* data are limited. This study aimed to improve methods for Hz quantification in tissues and to investigate the accumulation of Hz in different organs from mice infected with *Plasmodium* parasites with a varying degree of virulence.

**Methods:**

An improved method for extraction of Hz from tissues was elaborated and coupled to an optimized, quantitative, microtiter plate-based luminescence assay with a high sensitivity. In addition, a technique for measuring Hz by semi-quantitative densitometry, applicable on transmitted light images, was developed. The methods were applied to measure Hz in various organs of C57BL/6 J mice infected with *Plasmodium berghei* ANKA, *P. berghei* NK65 or *Plasmodium chabaudi* AS. The used statistical methods were the Mann–Whitney *U* test and Pearsons correlation analysis.

**Results:**

Most Hz was detected in livers and spleens, lower levels in lungs and kidneys, whereas sub-nanomolar amounts were observed in brains and hearts from infected mice, irrespectively of the parasite strain used. Furthermore, total Hz contents correlated with peripheral parasitaemia and were significantly higher in mice with a lethal *P. berghei* ANKA or *P. berghei* NK65-infection than in mice with a self-resolving *P. chabaudi* AS-infection, despite similar peripheral parasitaemia levels.

**Conclusions:**

The developed techniques were useful to quantify Hz in different organs with a high reproducibility and sensitivity. An organ-specific Hz deposition pattern was found and was independent of the parasite strain used. Highest Hz levels were identified in mice infected with lethal parasite strains suggesting that Hz accumulation in tissues is associated with malaria-related mortality.

## Background

With growing world populations in tropical countries, every year more people become exposed to malaria. As many areas of endemic malaria transmission overlap with regions of poverty, direct and indirect burdens of this infectious disease are important. In particular, a small percentage of the malaria-infected patients develop life-threatening complications such as severe malarial anaemia, encephalopathy, placental malaria or respiratory problems, even if they have similar peripheral parasitaemia levels compared to patients with mild or asymptomatic malaria [1]. Efficient adjunct therapies against these immunopathological complications are still not available. Therefore, studying the mechanisms of disease development in severe malaria is paramount.

Inside the red blood cell (RBC), almost 80% of the haemoglobin (Hb) is degraded, which means that high amounts of toxic haem (which is rapidly oxidized to haemin) are liberated in the food vacuole of the parasite capable of generating reactive oxygen species and damaging cell membranes and proteins [2,3]. As a detoxification mechanism, the parasite biocrystallizes the haemin molecules into insoluble haemozoin (Hz) crystals or malaria pigment [4,5]. When the schizont ruptures, Hz is released into the circulation and is rapidly removed by phagocytes inside several organs. Multiple *in vitro* and *ex vivo* pro-inflammatory and immunosuppressive effects of Hz have been described (reviewed in [4-7]). However, few *in vivo* data about the effects of Hz on the immune system exist. As large amounts of Hz are produced during infection and accumulate inside multiple organs, Hz may be important for the progress towards malaria-associated pathologies. This hypothesis is further strengthened by the fact that abundant Hz has been observed in brains [8-10] and placentas [11-13] from malaria patients with cerebral and placental complications, respectively. In addition, Hz was detected in brains of mice with cerebral symptoms [14,15] and in lungs of mice with malaria-associated acute respiratory distress syndrome (MA-ARDS) [16].

Experimental mouse models offer useful tools to study malaria-related disease mechanisms. Depending on the mouse-parasite combination, different aspects of human malaria can be mimicked and investigated, even if these models are not exact replicas and should thus be extrapolated with caution. In this study, C57BL/6 J mice were infected with three different parasite strains with a varying degree of pathogenicity. The *Plasmodium berghei* ANKA *(Pb*ANKA*)* parasite induces typical symptoms of cerebral malaria (CM), such as paralysis or coma and mice succumb within seven to nine days. In this mouse model, the pathology critically depends on activation of leukocytes, including CD8^+^ T cells, and a local inflammatory reaction [1]. Although sequestration of *Plasmodium falciparum* in the brain is strongly associated with CM in patients, it is unclear whether specific cyto-adherence of *Pb*ANKA occurs in the brain [17]. However, local parasite accumulation in the brain is thought to be an important feature of this model [18]. Mice infected with *Plasmodium berghei* NK65 *(Pb*NK65*)* do not develop such an encephalopathy but rather die from severe respiratory problems between nine and eleven days post-infection [16]. This respiratory pathology closely resembles human MA-ARDS, as in both mice and patients leukocytes (predominantly macrophages and lymphocytes) and infected RBCs (iRBCs) accumulate in the lungs, resulting in the disruption of endothelial barriers, severe edema and intra-alveolar hyaline membrane formation [16,19,20]. *Plasmodium chabaudi* AS (*Pc*AS)-infections are self-limiting and the mice are able to recover. Moreover, C57BL/6 mice mount a protective immune response against *Pc*AS-parasites mediated by phagocytes, CD4^+^ T cells and specific antibodies, which is very similar to the immune response generated against *P. falciparum* in humans [21].

To investigate the organ-specific Hz deposition in these three mouse models, novel techniques are described in the present study to accurately quantify the Hz content in tissues. These methods were implemented to compare the amount of Hz between various organs and between similar organs from mice infected with parasites of different pathogenicity. Most Hz was found in livers and spleens. Far less Hz was detected in lungs and kidneys, whereas limited amounts of Hz were observed in hearts and brains, irrespectively of the parasite species. In addition, more Hz was found in mice infected with *Pb*NK65 or *Pb*ANKA compared with *Pc*AS-infected mice despite of similar peripheral parasitaemia levels.

## Methods

### Chemical products

All chemicals were purchased from Sigma-Aldrich (Bornem, Belgium), unless otherwise stated.

### Mice and parasites

C57BL/6 J mice (seven to nine weeks old) were obtained from Janvier (Le Genest-Saint-Isle, France) and placed in a conventional animal house with food and water *ad libitum*. Parasite growth in mice was supported by supplementing the drinking water with 0.375 mg/mL 4-amino benzoic acid. Mice were intraperitoneally infected with 10^4^ iRBCs by serial passage of tail vein blood obtained from a mouse that had been infected with one of the following parasite strains: *Pc*AS, *Pb*NK65 (kind gifts of the late Prof. D Walliker, University of Edinburgh, Scotland, UK) or *Pb*ANKA (Cl15CY1, a kind gift of Prof. C Jansse, Leiden University Medical Centre, The Netherlands). The percentage of infected erythrocytes in the peripheral blood was determined by microscopic analysis after Giemsa staining. Mice were sacrificed at the indicated time points after infection and blood was removed by heart puncture. Mice were perfused with Dulbecco’s phosphate-buffered saline (PBS) (Lonza, Verviers, Belgium) to remove circulating iRBCs from the organs. Livers, spleens, kidneys, lungs, hearts and brains were removed, weighed and stored at -80°C until further analysis. A part of the liver, spleen, lung and kidney was embedded in Tissue-Tek O.C.T. Compound (Sakura Finetek Europe B.V., Alphen aan den Rijn, The Netherlands) and frozen in liquid nitrogen-cooled isopentane for histological analysis. All experiments were approved by the local ethical committee (License LA121251, Belgium).

### Haemozoin quantification in organ cryosections by densitometric analysis

**Figure 1:**
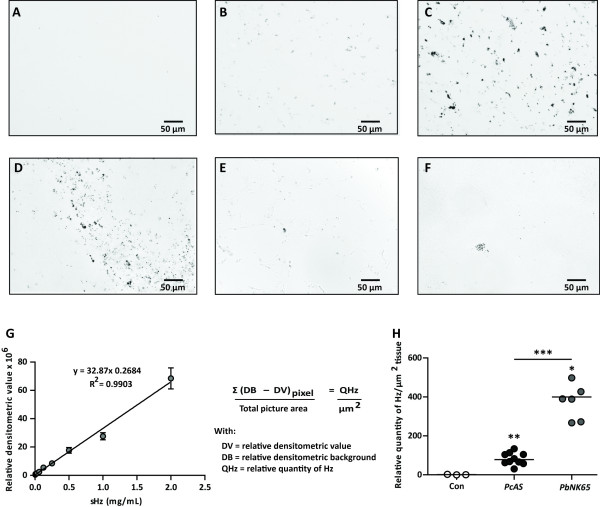
**Haemozoin detection by densitometric analysis.** Transmitted light images (grey scale) were taken from unstained 7 μm thick cryosections from livers of uninfected mice (**A**) and from mice infected with *Pc*AS (**B**) or *Pb*NK65 (**C**), from *Pc*AS-infected spleens (**D**), *Pb*NK65-infected lungs (**E**) and kidneys (**F**). In panel **G**, the relative densitometric value obtained from cryosections of gelatin blocks with different concentrations of sHz were analyzed and the formula used to calculate the relative quantity of Hz/μm^2^ is shown. In panel **H**, the relative Hz content was measured in liver sections from uninfected (Con), *Pc*AS and *Pb*NK65-infected mice ten days post-infection. Each dot represents the result from an individual mouse. Horizontal bars represent group medians and horizontal lines with asterisks on top indicate statistical comparisons between groups. Asterisks on top of data sets indicate statistical significances compared with the uninfected control group. * *p* < 0.05, ** *p* < 0.01 and *** *p* < 0.001

Cryosections with a thickness of 7 μm were prepared from frozen livers, spleens, lungs and kidneys and imaged by light microscopy. Transmitted light images were taken through a 20x/0.8 Plan-Apochromat objective of an Axiovert 200 M microscope equipped with an AxioCam MRm camera (Zeiss, Göttingen, Germany). For Hz quantification on liver sections, images were obtained from two rows of three consecutive fields. The densitometric analysis was performed with the AxioVision 4.6 software with a home-written script and the relative quantity of Hz/μm^2^ was calculated with the formula as shown in Figure 1G. The densitometric value (DV) of a pixel reflects the intensity of transmitted light at this position in the section. The densitometric background (DB) was determined in each picture by calculating the mean DV of all pixels with a DV above an empirically determined threshold. To test the linearity of the densitometric method to measure Hz on cryosections, gelatin blocks containing different concentrations of synthetic Hz (sHz) were analysed. sHz was prepared as described previously [22] and was homogenized and added in different concentrations to a 10% gelatin-PBS solution at 37°C in a 24-well plate. Upon solidification on ice (to prevent sedimentation of sHz), sHz-containing gelatin blocks were cut out of the wells, embedded in O.C.T and frozen at -80°C. For each concentration, five to six images were analysed from one section/sHz block and this was done for two blocks.

### Haem quantification by colorimetric analysis and haem-enhanced luminescence

The Fe-ions in the haemin molecules that constitute the Hz crystal are in the oxidized state (Fe^3+^). Therefore, a dilution series of a haematin stock solution (10 μM – 1.2 nM), prepared by dissolving haemin in 100 mM NaOH, 2% SDS and 3 mM EDTA, was used as a standard to compare methods. In an alkaline environment, haematin produces a brown colour measurable spectrophotometrically (Biotech Powerwave XS) at 405 nm [23]. Background absorbance was evaluated from a blank sample and subtracted from the measurements. The method for quantification by luminescence was based on the method of Schwarzer *et al.*[24] and was optimized for working in a 96-well plate and modified according to Yuan *et al.*[25]. Different concentrations of haematin were added in 96-well plates suitable for luminescence (Perkin Elmer, Waltham, MA, USA) and diluted in a solution containing NaOH and Na_2_CO_3_ (four volumes of 100 mM NaOH, 2% SDS and 3 mM EDTA and one volume of 1 M Na_2_CO_3_, pH 10.4) (final volume 50 μL). After addition of 100 μL luminol (100 μg/mL 3-aminophtalhydrazide) and 100 μL of peroxide (7% *tert*-butyl hydroperoxide), both dissolved in the NaOH/Na_2_CO_3_-solution, light emitted in the presence of Fe^3+^ (present in the haematin core) was measured during one second using a Thermo Luminoskan Ascent apparatus. Peroxide catalysis into oxygen by Fe^3+^ is a fast reaction. Therefore, special care was taken to keep the time between the addition of the peroxide and the luminescence measurements minimal and as similar as possible between the different wells (maximum time deviation between individual wells was eight seconds). A sigmoidal relationship between the haematin concentration and the luminescence (events/sec) was obtained. Background luminescence was evaluated from a blank sample and subtracted from the measurements. The above method for haem-enhanced luminescence was used for all measurements unless differently stated. The time-dependence of the luminescence signal was measured with 125 nM haematin in duplicate every ten seconds for eight minutes during a kinetic reading without any other samples in the plate to allow fast repetitive reading of these two wells. A lag-time of twelve seconds existed between the addition of the peroxide and the start of the measurement, which was partly attributable to a shaking step.

### Haemozoin determination in tissues and trophozoites

To extract haemozoin from perfused mouse tissues, approximately 30 – 60 mg (liver, spleen, kidney or lung), half brain or a full heart were homogenized with the Precellys Lysing Kit (VWR, Leuven, Belgium) in minimum five volumes of a solution containing 50 mM Tris/HCl pH 8.0, 5 mM CaCl_2_, 50 mM NaCl and 1% Triton X-100. The homogenate was supplemented with 1% Proteinase K and incubated overnight at 37°C. The next day the proteinase K digest was sonicated (VialTweeter, Hielscher Ultrasonics GmbH, Teltow, Germany) for 1 min (10 W, pulse 0.5 sec) and centrifuged at 11,000 x g for 45 min. The supernatant was discarded and the pellet was washed three times in 100 mM NaHCO_3_, pH 9.0 and 2% SDS with subsequent sonication and centrifugation for 30 min to remove degraded tissue, free haem and Hb. After the third wash, the pellet (Hz) was dissolved and sonicated in 100 mM NaOH, 2% SDS and 3 mM EDTA to form haematin and centrifuged to pellet any remaining insoluble material. To confirm that the isolated material was indeed Hz, it was examined for its birefringence character. For this purpose, isolated Hz that was washed three times as described above, was subsequently washed in distilled H_2_O to remove the salts, smeared on a glass slide and monitored by polarized light microscopy with a 40x/1.3 oil EC Plan-Neofluar objective of an Axiovert 200 M microscope.

To isolate Hz from trophozoites, heparinized blood was obtained by heart puncture from *Pb*NK65-infected mice and trophozoites were cultivated *ex vivo* overnight and harvested as described [26]. After determination of the total red cell number and the percentage of iRBCs, Hz was extracted from the cells as described above but without proteinase K treatment and subsequently dissolved as described.

The extracted Hz was measured in different dilutions with the above-mentioned protocol for haem quantification by luminescence. A dilution series of haematin (10 μM – 1.2 nM) was used as a standard. The unknown Hz concentration was calculated from the calibration curve of the haematin concentration (nM) *versus* luminescence (events/sec). Background luminescence was evaluated from a blank sample and subtracted from the measurements. The amount of Hz (fmol or pmol haematin/mg tissue) was multiplied with the total weight of the concerning organ and expressed as pmol or nmol haematin/organ. An accuracy limit was estimated for each organ separately.

### Statistical analysis

*P*-values for the differences between two groups were calculated with the Mann–Whitney *U*-test, using the GraphPad Prism software (GraphPad Software, San Diego, CA, USA). The same software was used for linear regression analyses and for calculating Spearman correlation coefficients. The slopes of the individual regression lines of the different groups were compared online [27]. A *p*-value less than 0.05 (*p* < 0.05) was taken as statistically significant.

## Results

### Haemozoin detection by densitometric analysis

Pigment distribution was investigated on unstained cryosections from various organs of mice infected with *Pb*NK65 or *Pc*AS and monitored with light microscopy. Hz was observed on transmitted light images from infected mouse organs as brown pigments and was absent in organ sections from uninfected mice as is shown for the liver (Figure 1A–F). The pigment was found equally distributed throughout the liver (Figure 1B–C), whereas in the spleen (Figure 1D), the lungs (Figure 1E) or the kidneys (Figure 1F), it was located in the red pulp, the interstitial tissue or clustered in presumably the glomeruli, respectively. Brains and hearts contained such low amounts of Hz that it was almost unnoticeable on unstained sections. To estimate the amount of Hz on organ cryosections, semi-quantitative densitometry with the AxioVision 4.6 software using a home-written script was applied. The linearity of this densitometric method was investigated on cryosections from gelatin-blocks with different concentrations of sHz. A broad linear relationship was found between the sHz concentration and the obtained relative densitometric value (Figure 1G). The detected Hz signal was converted into the relative quantity of Hz/μm^2^ tissue as calculated with the formula described in Figure 1G. This technique was used to measure Hz in liver cryosections from non-infected, *Pc*AS and *Pb*NK65-infected mice. Despite similar mean peripheral parasitaemia levels in both groups of mice (*Pc*AS 18%; *Pb*NK65 12.7%; *p* = 0.4), significantly more Hz/μm^2^ tissue was detected in livers of *Pb*NK65-infected mice compared with *Pc*AS-infected mice (Figure 1H). This technique combines *in-situ* information with relative quantification and can be used to measure Hz in organs with a high and evenly distributed Hz content such as livers. However, this method is poorly applicable for organs in which the pigment is not equally distributed, e.g. spleen, lungs and kidneys. In brains and hearts, the amounts of Hz were too low to be quantified by densitometry.

### Comparison of haem quantification by different techniques

**Figure 2:**
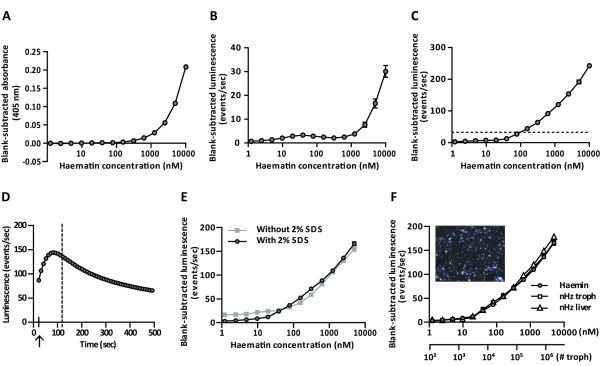
**Haem quantification by different techniques.** Different concentrations of haematin (10 μM – 1.2 nM) were used to compare the sensitivity of previously described techniques to quantify haem in a 96-well based format. In panel **A**, haematin was measured with a spectrophotometer at 405 nm (n = 4 for each concentration). Panel **B** shows the measurements of haematin concentrations by haem-enhanced luminescence with the same reagent conditions as described by Schwarzer and colleagues (20) for a cuvette-based system. Background absorbance/luminescence from a blank sample was subtracted from all the measurements. The adjusted chemo-luminescence protocol (100-fold higher luminol and peroxide concentrations buffered around pH 10.4) was used to measure the same concentrations of haematin in panel **C** (n = 8 for each concentration in B and C). The horizontal dashed line denotes the accuracy limit of the assay. The time-dependence of the luminescence signal and the effect of 2% SDS are displayed in panel **D** and **E**, respectively. The arrow in panel D denotes the start of the kinetic reading after an initial delay of twelve seconds, and the vertical dashed line indicates the moment of luminescence detection during the experimental readings. In panel **F**, natural Hz was isolated from trophozoites (troph) and livers, its concentration was determined by absorption at 405 nm and the concentration-dependence of the haem-enhanced luminescence was measured. Inset of panel F is a picture demonstrating the birefringence of the isolated Hz. The amount of Hz/trophozoite was calculated and the corresponding number of trophozoites (# troph) is integrated in the figure as a second X-axis

Because the densitometric analysis is not suitable to quantify Hz in tissue sections from all organs, a more sensitive technique was designed to determine Hz in organ extracts. Several methods for quantifying Hz in blood are described in literature, including a colorimetric [23] and a chemo-luminescence assay [24]. These two techniques were adapted to a 96-well plate format and the sensitivity was compared by measuring different concentrations of haematin produced when haemin or Hz is dissolved in an alkaline environment. A sigmoidal relationship was obtained between the haematin concentration and the blank-subtracted absorbance at 405 nm (Figure 2A) or the blank-subtracted luminescence with the reagent concentrations used as described for a cuvette-based system by Schwarzer *et al.*[24] (Figure 2B). In the 96-well plate format adapted for measuring absorbance or luminescence with a plate reader, both techniques detected the presence of haematin starting from a minimal concentration of 1 μM. To improve the sensitivity, the chemo-luminescent assay was adjusted by stabilizing the pH around 10.4 and by raising the concentrations of luminol and peroxide 100-fold. In this way, a detection limit around 100 nM was obtained (Figure 2C). Furthermore, the time-dependence of the luminescence signal was examined by measuring the emitted light of a single concentration during a kinetic measurement. With the described protocol for haem-enhanced luminescence in a 96-well plate format, emitted light was measured when the luminescence signal was almost maximal (Figure 2D). The sensitivity of this 96-well plate-based assay is significantly lower than the sensitivity of the cuvette-based method described by Schwarzer *et al.*[24]. However, the current 96-well based method has the advantage of a higher throughput and the sensitivity appeared sufficient to measure Hz in organ extracts. To extract Hz from organs, the method described by Sullivan *et al.*[15] was optimized. The most important modification was the addition of an overnight proteinase K digestion step that eliminated high background signals e.g. in lung samples. After several wash steps to remove any free haemin, the Hz crystals were converted to free haematin by dissolving in a strong alkaline environment, so that the haematin concentration could be measured. Since the extraction procedure involved washing steps in the presence of SDS, possible quenching of the emitted light by SDS was investigated and had no effect on the luminescence catalyzed by haematin-Fe^3+^ (Figure 2E).

As a final check up for the appropriateness of the assay for quantifying natural Hz, the concentration dependence of the haem-enhanced luminescence measured with the plate reader was studied with natural Hz purified from trophozoites and from the liver. As shown in Figure 2F, haemin and Hz derived from trophozoites or livers behaved similarly in the assay confirming the suitability of using haemin as a standard for deducing the Hz concentration in the samples. In addition, as birefringence is a typical feature of Hz [28], Hz isolated from the liver was spread on a glass slide and monitored by polarized light microscopy. This confirmed that the isolated material consisted mainly of Hz (Figure 2F, inset).

### Quantitative analysis of haemozoin in various organs of mice infected with different parasite species

**Figure 3:**
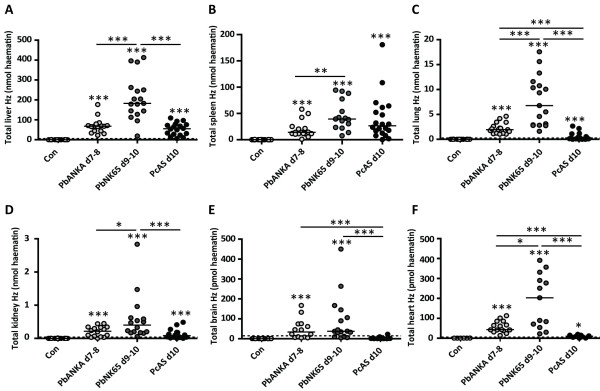
**Quantification of haemozoin in tissues.** C57BL/6 J mice were infected with 10^4^*Pb*ANKA, *Pb*NK65 or *Pc*AS parasites or were left uninfected (Con). At the indicated time intervals after infection, mice were dissected after heart puncture and perfusion. Extracted Hz from 30 – 60 mg tissue of livers (**A**), spleens (**B**), lungs (**C**) and kidneys (**D**), from half brains (**E**) and from whole hearts (**F**) were quantified by haem-enhanced luminescence and expressed as nmol haematin/organ (liver, spleen, lungs and kidneys) or pmol haematin/organ (brain and heart). Each group consisted of 15 to 20 mice, with each dot indicating individual data points. Horizontal dashed lines were used to denote the accuracy limit of the assay for each organ separately. Horizontal bars represent group medians and horizontal lines with asterisks on top indicate statistical comparisons between groups. Asterisks on top of data sets indicate statistical significances compared with the uninfected control group. * *p* < 0.05, ** *p* < 0.01 and *** *p* < 0.001

The optimized haem-enhanced luminescence technique was used to study differences in the amount of Hz between various organs and between the same organs of mice infected with parasites of different pathogenicity (*Pb*ANKA, *Pb*NK65 or *Pc*AS)*.* Mice were sacrificed at the indicated times post-infection and perfused systemically. Even though no difference was found in the amount of Hz before and after perfusion ( Additional file 1), it seemed more reasonable to apply perfusion on all samples tested. In this way, there could be no doubt that the detected Hz represented organ-trapped Hz and not Hz present in the circulation. Quantification of the total amount of Hz per organ revealed that most Hz was present in livers followed by spleens (Figure 3A–B). Far less Hz was detected in lungs and kidneys (Figure 3C–D), whereas subnanomolar levels of Hz were found in brains and hearts, irrespectively of the parasite species used (Figure 3E–F). Livers, lungs, kidneys and hearts from *Pb*NK65-infected mice nine to ten days post-infection contained significantly more Hz compared with the same organs from *Pb*ANKA-infected mice seven to eight days post-infection or *Pc*AS-infected mice ten days post-infection, even though livers of *Pc*AS-infected mice were significantly larger (*p* < 0.0001 for liver weights between *Pc*AS d10 and *Pb*NK65 d9-10 and between *Pc*AS d10 and *Pb*ANKA d7–8). In addition, lungs and hearts from *Pb*ANKA-infected mice seven to eight days post-infection had significantly more Hz compared with the same organs from *Pc*AS-infected mice after ten days of infection. The total amount of Hz was similar in spleens of *Pc*AS and *Pb*NK65-infected mice ten days post-infection (Figure 3B), although the amount of Hz/mg spleen tissue was six-fold lower in mice infected with *Pc*AS compared to *Pb*NK65 (median value was 1164.4 pmol haematin/mg spleen for *Pb*NK65 and 199.9 pmol haematin/mg spleen for *Pc*AS; *p* < 0.0001). This was compensated by the three- to four-fold larger spleen size in *Pc*AS-infected mice (*p* < 0.0001). Furthermore, similar amounts of Hz were observed in brains from *Pb*NK65 and *Pb*ANKA-infected mice, whereas less Hz was found in brains of *Pc*AS-infected mice.

### Different total haemozoin levels in *Pc*AS-infected mice and *Plasmodium berghei*-infected mice

**Figure 4:**
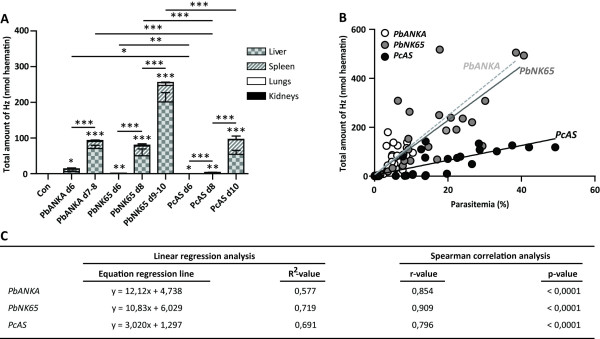
***Pc*AS-infected mice contain less total Hz than*Plasmodium berghei*-infected mice.** The total amount of Hz (nmol haematin)/mouse was estimated from the sum of the Hz content in the individual organs. Panel **A** shows the average amount of Hz found in C57BL/6 J mice infected with *Pb*ANKA, *Pb*NK65 or *Pc*AS on different time points post-infection, with the contribution of each organ to the total amount of Hz ± SEM. Asterisks on top of data sets indicate the statistical significances compared with the uninfected control group. Horizontal lines with asterisks on top indicate statistical differences between infected groups. * *p* < 0.05, ** *p* < 0.01 and *** *p* < 0.001. In panel **B**, the total amount of Hz (nmol haematin) was correlated with the level of parasitaemia (%) for each parasite strain separately and the individual regression lines are shown. The regression line of *Pb*ANKA was prolonged under the form of a hatched line to better distinguish it from the *Pb*NK65 line. The equation of the regression lines and the R^2^-values, together with Spearman r-values and *p*-values are shown in panel **C**

As brains and hearts contained only subnanomolar amounts of Hz, individual total Hz levels were calculated by making the sum of organ-specific Hz levels from liver, spleen, lungs and kidneys. Mice infected with parasites of the *P. berghei* strains contained significantly more Hz than mice infected with *Pc*AS-parasites (Figure 4A). Moreover, the total amounts of Hz correlated with the peripheral parasitaemia levels for all parasite strains (Figure 4B and C). *Pb*ANKA and *Pb*NK65-parasites had a similar Hz-production pattern, i.e. comparable amounts of Hz at similar peripheral parasitaemia levels. In contrast, *Pc*AS-infected mice seemed to have significantly less Hz in relation with their parasitaemia compared with *Pb*ANKA or *Pb*NK65 as indicated by the lower slope on the regression curve. The difference between the slopes of the regression lines was significantly different between *Pb*NK65 and *Pc*AS-infected mice (*p* < 0.0001).

## Discussion

The first observations of black pigment in necroptic spleens and brains go back to the 18^th^ Century (reviewed in [5]). About 130 years later, a publication mentioned brown-grey colourations of brain, spleen and liver, which turned out to arise from pigment deposition. At first believed to be melatonin, it was later linked to a parasitic disease. Presently, many *in vitro* and *ex vivo* immunomodulating effects have been ascribed to Hz [4-7]. However, data about the fate and properties of Hz in the *in vivo* situation are still scarce. Hz is released in the circulation in considerable amounts after schizont rupture where it may interact with a whole range of different cell types. The majority of the liberated Hz is presumably captured and phagocytosed by circulating and tissue resident monocytes/macrophages in which it can persist for a long time. In this way, Hz may be capable of causing considerable inflammation that might progress to tissue injury. In this study, techniques for sensitively quantifying the amount of Hz in tissues were examined and the organ-specific Hz content was compared between parasite species with a varying degree of pathogenicity.

As Hz crystals were observed on unstained cryosections from livers, spleens, lungs and kidneys, a technique for estimating the amount of Hz in these sections by densitometric analysis was developed. As Hz is proportionally distributed throughout the liver, the estimation of the amount of Hz by densitometry was quite reliable. In other organs, however, Hz was found in specific structures such as the red pulp in the spleen, the interstitial tissue in the lungs or presumably the glomeruli in the kidneys. This may in part be attributed to the differential localization of tissue-resident phagocytes. This implies that Hz distribution is a confounding factor for the accuracy of the Hz measurements on organ cryosections by densitometry. In addition, this technique is time-consuming, labour-intensive, semi-quantitative and not suitable for organs with a low Hz content and was thus not further explored. Therefore, a more sensitive, analytical and quantitative method for determining the Hz content in tissues was investigated. To isolate Hz from organs, a protocol described by Sullivan and colleagues [15] was modified. The main adaptation was the digestion of the homogenates with proteinase K. This digestion eliminated high background signals, which were presumably due to the binding of Hb to otherwise insoluble extracellular matrix components. Upon conversion of the isolated Hz into soluble haematin, a chemo-luminescence assay was used for quantification. This assay was based on the method of Schwarzer *et al.*[24] and adapted to microtiter plate format. The obtained sensitivity with the optimized protocol was lower compared with the haem-enhanced luminescence assay described by Schwarzer *et al*. This was not due to quenching of the luminescence signal by SDS nor was it caused by the altered time frame during which the emitted light was measured (two seconds/sample *versus* approximately ninety six seconds/plate), but probably originated from the use of different luminescence detector systems (cuvette system *versus* microplate reader). Nevertheless, the microplate-adjusted approach offers the advantage of measuring several samples in varying concentrations simultaneously with a sensitivity that is optimal for the quantification of Hz in malaria-infected organs.

As an application, the distribution of Hz throughout the body of infected mice was studied and compared between diverse parasite strains with varying pathogenicity. Sullivan and colleagues already quantified the Hz content in brains, livers and spleens of mice [15,29] and in human placentas [11], but no detailed comparison between organs and between parasite species was described. Almost 95% of the total pool of Hz was found in livers and spleens. This was expected as large volumes of blood are filtered through these organs and both contain a vast population of tissue-resident monocytes/macrophages capable of rapidly removing the crystalline material from the circulation by means of phagocytosis. It was also important to consider the liver and spleen sizes when determining the total Hz amounts, as these sizes evolve in a different way during infection with different parasites (i.e. induction of hepatosplenomegaly by *Pc*AS). As the absolute Hz concentration in the organs could be determined by the luminescence assay, this was easily taken into account by multiplication with the organ weights.

Furthermore, substantial amounts of Hz were detected in lungs of malaria-infected mice. In a new mouse model of MA-ARDS [16], considerable amounts of Hz were observed on histological sections of the lungs. By quantifying the Hz content in the lungs, significantly higher Hz levels were validated in lungs from *P. berghei*-infected mice (lung pathology) compared to *Pc*AS-infected mice (no lung pathology), indicating that Hz may have a role in the development of malaria-associated lung disease.

Low but detectable amounts of Hz were found in kidneys, hearts and brains of malaria-infected mice. Most Hz was detected in kidneys and hearts from *Pb*NK65-infected mice ten days post-infection compared with *Pb*ANKA and *Pc*AS-infected mice seven to eight and ten days post-infection, respectively. However, a different pattern was observed in the brains, i.e. Hz was undetectable in brains from *Pc*AS-infected mice whereas similar amounts of Hz were detected in brains of *Pb*NK65 and *Pb*ANKA-infected mice. A possible explanation for this difference is their diverse parasite synchronicity. At the moment of sacrificing the mice and organ removal, the *Pc*AS-parasites in the circulation were all in the ring and young trophozoite stage. As these developmental stages do not yet contain abundant Hz [3,10], it seemed reasonable that Hz was not detected in brains from mice infected with this parasite species. It is also possible that no Hz was detected because *Pc*AS-parasites may not sequester in the brains as is the case for *Plasmodium vivax*-infected erythrocytes [10]. On the contrary, several developmental stages of *P. berghei* parasites are found in the circulation simultaneously and accumulation of *P. berghei* in the brain is still a debated issue. The observation of similar brain Hz contents in *Pb*ANKA and *Pb*NK65-infected mice cannot be explained by their parasitaemia levels as significantly higher parasitaemias were found in mice that were infected with *Pb*NK65 than in mice infected with *Pb*ANKA. The data however do suggest that Hz as such is not sufficient for the development of this immunopathology as *Pb*NK65-infected C57BL/6 J mice do not develop cerebral complications [16]. These data are in contrast with data from Coban *et al.*[14] and Sullivan *et al.*[15] who found that brains from mice with cerebral pathology contained more Hz than healthy brains from infected mice. However, this may be explained by differences in the timing of analysis after infection and in the mouse or parasite strains used in the studies.

Organ-trapped Hz may originate from two sources. As free Hz is rapidly removed from the circulation, it is found either inside phagocytes or inside cyto-adhering iRBCs along the endothelial lining of the organs’ microvasculature. Systemic perfusion removes circulating iRBCs but not sequestering iRBCs or Hz inside resident phagocytes, although inadequate perfusion can result from obstruction due to organ-specific cyto-adherence and haemorrhages. Furthermore, it is still not completely clarified if sequestration by murine malaria parasites occurs and which organs are the main targets. Local parasite accumulation has been demonstrated in brains and lungs of *Pb*ANKA-infected mice suffering from cerebral symptoms [18,30], but no reports exist on *Pb*NK65-parasite sequestration.

After calculating the total amount of Hz in the mice, it was found that *Pb*ANKA and *Pb*NK65-infected mice contained similar amounts of Hz at comparable parasitaemia levels. This suggests that both parasites produced similar amounts of Hz, or that their schizonts presumably consumed comparable amounts of Hb. On the contrary, lower amounts of Hz were retrieved in *Pc*AS-infected mice despite of similar peripheral parasitaemia. Several explanations can be given for this finding. *Pc*AS-parasites may produce less Hz, e.g. by digesting less Hb or by using other haem detoxification mechanisms (transport of haem out of the food vacuole or anti-oxidative defense mechanisms of the parasite) or *Pc*AS Hz could be more easily degraded. Interestingly, Noland *et al.*[31] demonstrated that Hz crystals from different *Plasmodium* species have different shapes and dimensions, supporting the notion that Hz from different species may have different properties. In addition, Hz contents are variable in RBC infected with different *Plasmodium falciparum* strains [32].

Another possibility is that peripheral parasitaemia, estimated by counting the percentage of iRBCs by microscopic analysis of Giemsa-stained blood smears, are not a true reflection of the total parasite biomass as they do not take sequestered parasites into account. Consequently, it is possible that *Pc*AS-infected mice contain less Hz because of lower total parasite burdens. These observations may well translate to the situation in human malaria, where various parasite species have different degrees of virulence. Total parasite biomass in *P. falciparum* infections is higher than peripheral parasitaemia levels and the difference between these two parameters increases with disease severity [33]. Similarly, Hz-containing peripheral leukocytes are a marker for disease severity [34-36], and accumulation of Hz in brain micro-vessels is associated with a subtype of cerebral malaria [37]. No data are available yet about total parasite burdens in *P. vivax*-infections and it is still questionable if *P. vivax*-iRBCs can adhere to the endothelial micro-vascular lining. However, cytoadhesion of *P. vivax*-infected erythrocytes was demonstrated *in vitro*[38] and, despite of the absence of sequestration in the brain [10], it was hypothesized that parasitized RBCs might sequester in lungs from patients with *P. vivax* malaria [39]. Similarly, very little knowledge exists on the role of Hz in *P. vivax* infections.

Besides differences in pathogenicity, another interesting difference between *P. berghei* and *Pc*AS is that *Pc*AS can be cleared from the circulation in several mouse strains, including C57BL/6 mice, whereas *Pb*ANKA and *Pb*NK65 cannot. The amounts of Hz produced by these parasites may also contribute to these differences, as Hz is known to suppress macrophage activity *in vitro*[40] and *in vivo*[41]. Interestingly, Spaccapelo *et al.* found that plasmepsin 4-deficient *Pb*ANKA-parasites, which produce less Hz, cause less immunopathology and are more easily cleared by some mouse strains [42].

## Conclusions

This paper describes newly developed and improved methods for sensitive Hz quantification in mouse organs. Different amounts of Hz were detected in the analysed organs and total Hz contents were highest in mice that were infected with lethal parasite strains. Therefore, it is clear that these techniques will be valuable in the investigation of a possible relationship between Hz and organ-specific malaria pathologies.

## Abbreviations

CM: Cerebral malaria; DB: Densitometric background; DV: Densitometric value; Hb: Haemoglobin; Hz: Haemozoin; MA-ARDS: Malaria-associated acute respiratory distress syndrome; *Pb*ANKA: *Plasmodium berghei* ANKA; *Pb*NK65: *Plasmodium berghei* NK65; *Pc*AS: *Plasmodium chabaudi* AS; iRBC: Infected red blood cell; sHz: Synthetic haemozoin (beta-haematin).

## Competing interests

The authors declare that they have no competing interests.

## Authors’ contributions

KD participated in the design of the study, performed the animal experiments, optimized the luminescence technique, analysed and interpreted the data, performed the statistical analysis and drafted the manuscript. NL participated in Hz analysis. SN operated the microscope and designed the script for the densitometric analysis of Hz on pictures from organ cryosections. EM participated in optimizing the luminescence technique. GO participated in study design, manuscript writing and provided critical support. PVDS conceived and participated in the design of the study, optimized the method for densitometric analysis of Hz in cryosections and participated in interpreting the data and drafting the manuscript. All authors read and approved the final manuscript.

## Supplementary Material

Additional file 1 **Effect of perfusion on the organ-specific haemozoin content.** To investigate the effect of perfusion on the organ-specific haemozoin content, mice were infected intraperitoneally with 10^4^*Pb*NK65 parasites. Ten days later, mice were sacrificed, the right lung and kidney were pinched off, and the other organs were perfused with phosphate-buffered saline to remove circulating erythrocytes. The amount of Hz/mg tissue was quantified in both perfused and non-perfused lungs and kidneys with the modified 96-well plate based haem-enhanced luminescence assay. The total Hz content in lung and kidney was calculated by multiplying the amount of Hz/mg tissue with the organ weights. As shown in panel A and B, no difference was found in the amount of Hz in lungs and kidneys with or without perfusion. Nevertheless, perfusion was applied for all organs tested as it is conceptually more rational to remove circulating infected erythrocytes as a source of haemozoin. Each dot represents data of an individual mouse. (PDF 77 kb) Click here for file
