# Supplementary material for: Improved methods for haemozoin quantification in tissues yield organ-and parasite-specific information in malaria-infected mice
Source: Malar J. 2012 May 14;11:166. doi: 10.1186/1475-2875-11-166 (PMC3473299; doi:10.1186/1475-2875-11-166)
Supplement: Additional file 1 — Effect of perfusion on the organ-specific haemozoin content. To investigate the effect of perfusion on the organ-specific haemozoin content, mice were infected intraperitoneally with 104PbNK65 parasites. Ten days later, mice were sacrificed, the right lung and kidney were pinched off, and the other organs were perfused with phosphate-buffered saline to remove circulating erythrocytes. The amount of Hz/mg tissue was quantified in both perfused and non-perfused lungs and kidneys with the modified 96-well plate based haem-enhanced luminescence assay. The total Hz content in lung and kidney was calculated by multiplying the amount of Hz/mg tissue with the organ weights. As shown in panel A and B, no difference was found in the amount of Hz in lungs and kidneys with or without perfusion. Nevertheless, perfusion was applied for all organs tested as it is conceptually more rational to remove circulating infected erythrocytes as a source of haemozoin. Each dot represents data of an individual mouse. (PDF 77 kb) [file 1475-2875-11-166-S1.pdf]

Additional file 1: Effect of perfusion on the organ-specific haemozoin content

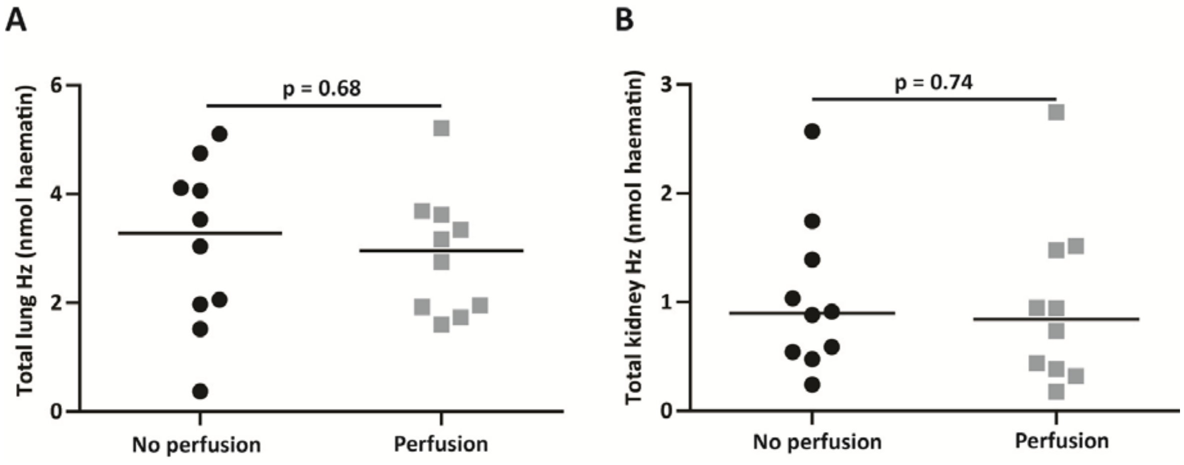

To investigate the effect of perfusion on the organ-specific haemozoin content, mice were infected intraperitoneally with 10^4 *PbNK65* parasites. Ten days later, mice were sacrificed, the right lung and kidney were pinched off, and the other organs were perfused with phosphate-buffered saline to remove circulating erythrocytes. The amount of Hz/mg tissue was quantified in both perfused and non-perfused lungs and kidneys with the modified 96-well plate based haem-enhanced luminescence assay. The total Hz content in lung and kidney was calculated by multiplying the amount of Hz/mg tissue with the organ weights. As shown in panel A and B, no difference was found in the amount of Hz in lungs and kidneys with or without perfusion. Nevertheless, perfusion was applied for all organs tested as it is conceptually more rational to remove circulating infected erythrocytes as a source of haemozoin. Each dot represents data of an individual mouse.
